# Evaluation of the Effects of an Undenatured Collagen Type-2-Based Nutraceutical (ARTHROSHINE^®^ HA²) on Recovery Time after TPLO in Dogs: A Prospective, Randomized Study with Objective Gait Analysis as the Primary Outcome Measure

**DOI:** 10.3390/ani14020298

**Published:** 2024-01-18

**Authors:** Maria Assies, Björn Berger, Bente Stegen, Thomas Rohwedder, Marcus Doherr, Peter Böttcher

**Affiliations:** 1 Fachtierärztliches Zentrum Dr. Berger, 26892 Heede, Germany; drbjoernberger@gmx.de (B.B.);; 2 Small Animal Clinic, Free University of Berlin, 14163 Berlin, Germany; thomas.rohwedder@fu-berlin.de (T.R.); peter.boettcher@fu-berlin.de (P.B.); 3 Institute for Veterinary Epidemiology and Biostatistics, Free University of Berlin, 14163 Berlin, Germany; marcus.doherr@fu-berlin.de

**Keywords:** TPLO, undenatured collagen type 2, gait analysis, LOAD

## Abstract

**Simple Summary:**

Undenatured collagen type 2 is considered a promising treatment for osteoarthritis in both humans and animals. Osteoarthritis, a degenerative joint disease, is often associated with cranial cruciate ligament rupture in dogs, which frequently necessitates Tibial Plateau Leveling Osteotomy (TPLO). Despite the generally successful outcomes of TPLO, dogs frequently experience postoperative pain and ongoing joint degeneration. To address these concerns, our study delved into the effects of oral supplementation with a high-concentration undenatured collagen type 2 dietary supplement (ARTHROSHINE^®^ HA²) post-TPLO surgery. Employing a prospective, randomized design, we utilized treadmill analysis and a validated owner questionnaire to objectively gauge the nutraceutical supplement’s influence on limb function, overall activity level, and pain. The results are highly encouraging. Gait analysis revealed a significant reduction in the duration of postoperative rehabilitation, enabling complete recovery within just 12 weeks after the procedure, compared to the 24 weeks required without the supplement. This research underscores the potential of undenatured collagen type 2 as an adjunctive therapy for alleviating lameness following TPLO surgery. Notably, subjective owner perceptions did not differ between the groups.

**Abstract:**

This randomized, prospective clinical trial investigates the impact of a novel undenatured collagen type 2 (T2NDC)-based nutraceutical, ARTHROSHINE^®^ HA² (AS), on postoperative rehabilitation following Tibial Plateau Leveling Osteotomy (TPLO) in 50 dogs with unilateral cranial cruciate ligament rupture (CCLR). The patients were randomly allocated to either group A, receiving AS once daily for 24 weeks post-TPLO surgery, or group B, without any supplementation. Frequency matching was applied to enhance group comparability. Assessment of outcomes included computerized gait analysis and a validated owner questionnaire. AS supplementation was well received, without any reported side effect. Consistently, patients in group A exhibited significantly higher peak vertical force values during all follow-up assessments. By the 12-week mark, gait analysis indicated a return to a physiological gait pattern in group A, while group B achieved this normalization only by the 24-week point. The administration of AS post-TPLO surgery demonstrates promise in enhancing limb function, leading to faster restoration of a physiological gait pattern. The inclusion of AS, a T2NDC-based nutraceutical, in the post-TPLO rehabilitation protocol may contribute to improved limb function and an expedited recovery, potentially facilitating a quicker return to normalcy. It is noteworthy that subjective owner perceptions did not differ between the two groups.

## 1. Introduction

Cranial cruciate ligament rupture (CCLR) is one of the most common orthopedic conditions in dogs, leading to various clinical symptoms such as joint swelling, effusion, stiffness, pain, and lameness, along with progressive osteoarthritis (OA) [1]. Tibial Plateau Leveling Osteotomy (TPLO) is a well-accepted and effective surgical treatment option for CCLR [2,3,4]. However, achieving full limb function following TPLO can take up to one year and the immediate and mid-term postoperative phase is characterized by pain, lameness, and disability [5,6,7]. Physiotherapy has been shown to effectively increase muscle mass as well as range of motion of the operated stifles, but physiotherapy is not effective at decreasing the degree of lameness or at increasing weight-bearing forces [8]. Prolonged administration of non-steroidal anti-inflammatory drugs is not a valuable strategy because of the well-known side effects [9,10,11,12,13,14].

Nutraceuticals, such as undenatured collagen type 2 (T2NDC), may not be associated with significant side effects, while positively impacting clinical function [15,16,17,18,19,20,21]. T2NDC is assumed to provide beneficial effects beyond improvement in range of motion and overall limb function by decreasing serum cartilage oligomeric matrix protein levels as well as interleukin-6 and matrix metalloproteinase-3 levels in synovial fluid, which are key components of the inflammatory cascade [15,20]. T2NDC also causes a down-regulation of the body’s immune response through a mechanism called “oral tolerization” taking place at the Peyer’s patches in the gut-associated lymphoid tissue of the small intestine [22,23,24,25]. Repeated oral administration of T2NDC has been shown to reduce T-cell attack on the cartilage structural protein collagen type 2 in injured joints, potentially targeting one aspect of degenerative joint disease [15].

ARTHROSHINE^®^ HA^2^ (AS; VetAthletics GmbH, Jülich, Germany) is a nutraceutical containing T2NDC in a high concentration (4.9% with 10.5% pure undenatured collagen type 2 in the finished product). Besides the high content of T2NDC, AS contains methylsulfonylmethane, yeast as a carrier, and hyaluronic acid. Further ingredients are manganese as a trace element of joint metabolism and vitamin E, which is reported to reduce the collagen catabolism by preventing protein oxidation when chondrocytes are submitted to an oxidative burst [26].

In the present study, we aimed to investigate the working hypothesis that the administration of AS leads to significant improvement in clinical outcomes and hastens the time to recover following TPLO for the treatment of unilateral CCLR in dogs.

## 2. Materials and Methods

### 2.1. Study Design

**Figure 1:**
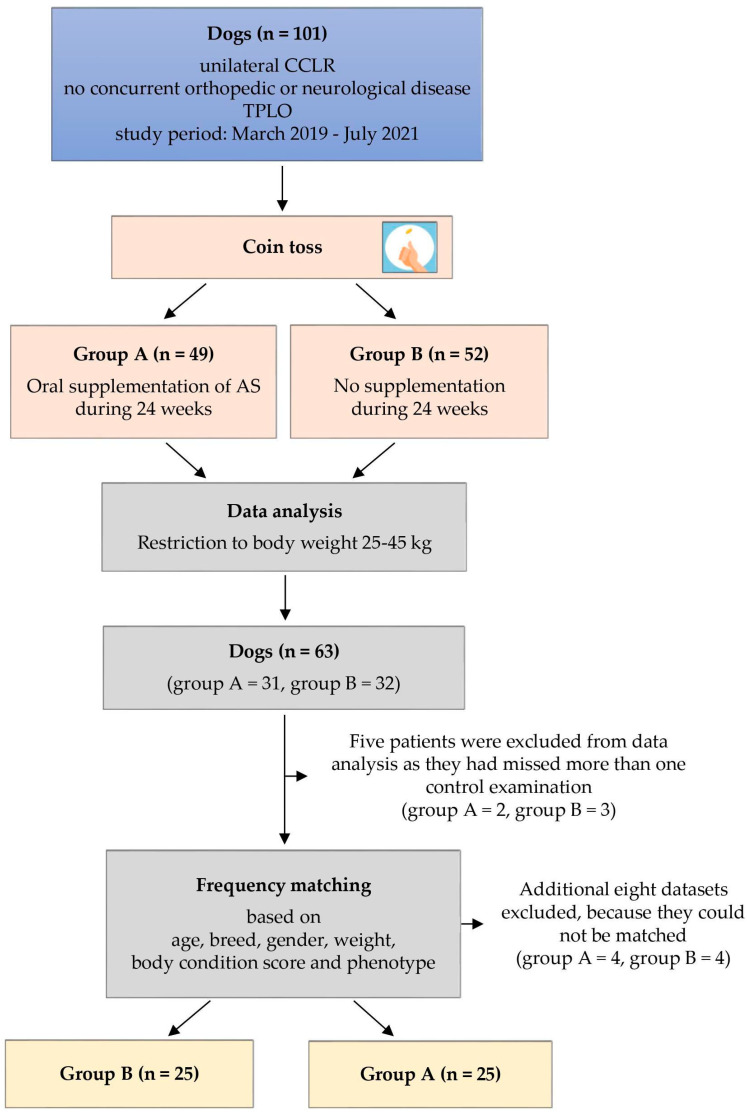
Flow chart providing a simplified representation of the study design. AS = ARTHROSHINE^®^HA^2^.

Between March 2019 and July 2021, a total of 101 client-owned dogs with unilateral partial or complete CCLR were enrolled in this prospective clinical trial, without regard to age, breed, gender, or weight (see Figure 1). The study was conducted in a veterinary specialist center for surgery and orthopedics. Dogs presenting with concurrent orthopedic or neurological conditions were excluded from the study following a comprehensive evaluation, which included a review of their medical history, clinical examinations, and radiographic assessments. After undergoing standard TPLO surgery, the patients were randomly assigned to either group A (*n* = 49) or group B (*n* = 52) using a random allocation method (coin tossing). Group A received daily oral AS supplementation following the manufacturer’s recommended dosage, which ranged from 4 to 6.1 mg/kg T2NDC based on body weight. Dosage guidelines were provided in measuring spoons per day (ms/d), with each ms containing 1.25 g of powder: <15 kg = 1 ms/d; 15–29 kg = 2 ms/d; 30–59 kg = 3 ms/d; >60 kg = 4 ms/d. Group B did not receive AS supplementation for the entire 24-week postoperative period. Besides AS supplementation, there were no differences in postoperative care between the two groups following TPLO surgery. Control examinations were scheduled at 6 weeks, 12 weeks, and 24 weeks after TPLO, and owner consent was obtained for every participant.

As medium to large breed dogs suffer from CCLR most frequently, only dogs with a body weight between 25 kg and 45 kg were included for the purpose of this study, resulting in 63 dogs (group A: *n* = 31, group B: *n* = 32) (see Figure 1) [27]. Five patients were excluded from data analysis as they had missed more than one control examination (group A: *n* = 2, group B: *n* = 3). To optimize comparability between the two groups, dogs were frequency-matched for age, breed, gender, weight, body condition score (BCS), and phenotype, with considerations including body type, leg length, and body height in a subjective manner. This matching process resulted in the withdrawal of four patients from each group, leaving 25 dogs in each group, with group A designated as the experimental group and group B as the control.

Treatment effects of AS were objectively quantified through computerized gait analysis at each recheck. Additionally, a subjective owner evaluation was conducted using the LOAD (Liverpool Osteoarthritis in Dogs) owner questionnaire, a validated clinical measurement tool [28]. Owners were also interviewed about acceptance of oral AS supplementation and any potential development of adverse effects at each recheck. As there are other studies lasting 24 weeks, the chosen study duration appeared to be appropriate [3,29]. Thus, the endpoint was set with the last follow-up examination after 24 weeks.

### 2.2. Surgery and Rehabilitation

Standard TPLO was carried out by one board-certified surgeon (B.B.), aiming for a postoperative TPA of 5° [30]. The osteotomy was fixed with a locking plate construct (Veterinary Orthopedic Implants, St. Augustine, FL, USA). Joint exploration was conducted via a cranio-medial mini-arthrotomy. Meniscal integrity was evaluated utilizing a 1 mm hook probe, without the aid of magnification or the use of any joint distractor. In instances where a medial and/or lateral meniscal tear was detected, appropriate treatment measures were implemented, involving the excision of the unstable tissue through partial meniscectomy. Partially torn but functional cranial cruciate ligament remnants were left undebrided.

Anesthesia regimen and medical aftercare were standardized with premedication of 0.025 mg/kg medetomidine IM and propofol IV to effect for induction of general anesthesia. At induction, patients received 0.5 mg/kg levomethadone IV, 0.2 mg/kg meloxicam SC, and 22 mg/kg cefazoline IV. Cefazoline was repeated every 90 min until wound closure. General anesthesia was maintained by controlled positive pressure ventilation with 2% isoflurane in a carrier gas mixture. Epidural anesthesia was performed with 0.2 mL/kg mepivacaine 2%.

Postoperatively, dogs received an oral medication of 4.4 mg/kg carprofen once daily and 22 mg/kg cefalexin twice daily for 10 days. Additional pain relief was provided by 3 mg/kg tramadol three times daily for 4 days. A bandage was applied for the initial 24 h following the surgery. On the second day, after removing the bandage, cold packs were applied three times for approximately 5–10 min each session. The dogs were discharged from the clinic about 32 h after the operation. Discharge management regime was the same for all TPLO-operated dogs. Each owner was given precise take-home instructions on how to behave in the following weeks. In all patients, the plaster was removed 3 days post-surgery and a wound check was performed after ten days. Nutrition was maintained at home as usual and exercise restriction with slowly increasing load bearing was applied equally to all study patients. Visiting physiotherapy and the administration of other nutritional supplements were not permitted for the duration of the study. The owners gave their verbal consent to comply with these regulations.

### 2.3. Computerized Gait Analysis

Computer-assisted gait analysis involved assessing peak vertical force (PVF) as a percentage of body weight (BW), calculating the difference in PVF (dPVF) between the operated and unaffected hind limb, as well as determining the symmetry index (SI) for PVF. Gait analysis, conducted on an instrumented treadmill, was performed before the surgery and at each subsequent follow-up appointment. The treadmill (CanidGait^®^, zebris Medical GmbH, Isny, Germany) had a tread surface area of 200 × 46 cm. Detection of forces was performed by high-precision, individually calibrated, capacitive pressure sensors. Two high-speed cameras were precisely synchronized with the treadmill’s pressure measuring sensors, allowing an assessment of the patient’s gait during gait analysis and after the session via laptop recording. At the commencement of the gait analysis, dogs were given a two-minute acclimation period on the treadmill before recording of six 15 s trials began. Gait velocity was adjusted to size, leg length, and condition of the patient preoperatively and was maintained throughout the study. An average speed of 3 to 3.5 km/h was selected via treadmill control panel and offered a proper gait at walking pace. A trial was considered valid if the dog was walking at a constant pace with no signs of distraction.

Regarding the interpretation of dPVF, it is important to note that minor asymmetry during kinetic gait analysis in dogs is generally considered a normal finding [31]. In fact, any value less than 3.2% is typically considered within the normal range [32]. This slight asymmetry is often attributed to trial-to-trial variation rather than representing a genuine biological difference between contralateral limbs.

Symmetry index was employed to assess load differences between the operated and unaffected hind limb, specifically calculated for PVF [33].

SI = ABS (200 × (X1 − X2)/(X1 + X2))

X1 = PVF of the right hind limb;

X2 = PVF of the left hind limb;

ABS = absolute value.

According to this equation, an SI of 0 indicates perfect restoration of hind limb symmetry. It is worth noting that, in dogs, normal gait symmetry often includes values ≤ 9, considering the presence of physiological gait imbalance [34,35].

For the evaluation of a return to normality, the data from dPVF and SI values were converted into a binary scale, utilizing the established thresholds of dPVF and SI: dPVF < 3.2%, SI ≤ 9 = 0 (healthy/balanced gait); dPVF ≥ 3.2%, SI > 9 = 1 (signs of lameness).

### 2.4. Data Analysis

All data were recorded in Microsoft Office Excel 2007 and analyzed using SPSS Statistics 27.0 (IBM Corp., Armonk, NY, USA). The normality of interval-scaled gait parameter values was assessed through the Kolmogorov–Smirnov and Shapiro–Wilk tests, along with normal probability (Q-Q) plots. These values were then reported as means with associated standard deviations. Group-wise comparisons were conducted using the 2-sample t-test, while categorical data were compared through cross-classification and Fisher’s exact test. A significance level of *p* ≤ 0.05 was applied for all tests.

## 3. Results

### 3.1. Comparability of Study Groups

Through frequency matching during the inclusion process, groups were made comparable, resulting in *p*-values ranging from *p* = 0.21 to *p* = 1.0 for factors including age, breed, gender, weight, BCS, and phenotype (details provided in Table 1). Frequency matching was only performed once, based on the data at time of surgery. A separate study evaluated both thigh circumference as an indicator of muscle mass and the extent of radiographic osteoarthritis at the time of enrollment. However, no significant differences were observed (*p* = 0.25, *p* = 0.65) [36].

Partial CCLR was diagnosed in seven dogs, with four dogs in group A and three dogs in group B (*p* = 0.69). Meniscal injuries necessitating subtotal meniscectomy of the caudal horn of the medial meniscus were documented in 18 cases, with eight dogs in group A and ten dogs in group B (*p* = 0.57). No lateral meniscal tears nor complex or degenerative tears of the medial meniscus were diagnosed. Out of the 50 CCLRs, 30 occurred on the left and 20 on the right stifle, with 15 and 10 cases in each group, respectively.

Number of complications was equally distributed between groups, occurring in ten stifles throughout the study period of 24 weeks, being all minor and not requiring surgical revision. Fibular fractures were documented radiographically at the six-week recheck in six stifles (group A: *n* = 3, group B: *n* = 3; *p* = 1.0). At that point, advanced callus formation was evident, which progressed to bony union at 12 weeks. Patellar ligament tendinitis was perceived in two stifles (group A: *n* = 1, group B: *n* = 1; *p* = 1.0), being associated with pain on direct palpation of the swollen part of the ligament. Treatment consisted of two sessions of focused extracorporeal shock wave therapy (frequency level: 6 Hz, intensity level: 6, impulses: 2000, PiezoWave^2^, WOLF ELvation Medical, Kieselbronn, Germany), resulting in resolution of clinical symptoms within 7 days. Fracture at the apex patellae was evident in another two cases (group A: *n* = 1, group B: *n* = 1; *p* = 1.0), appearing to be asymptomatic in both.

Over the study period of 24 weeks, we failed to acquire 14 recheck evaluations. At week six, datasets of 48 of the enrolled 50 dogs were available (group A: *n* = 24, group B: *n* = 24) and for the 12-week time point, 47 datasets were available (group A: *n* = 23, group B: *n* = 24; *p* = 0.56). Both times, poor owner compliance was the reason. For the final recheck at 24 weeks, only data from 41 dogs were available (group A: *n* = 24; group B: *n* = 17, *p* = 0.01). Four owners, all from group B, did not attend the follow-up because of lack of motivation, three dogs had died, also from group B, and two dogs (group A: *n* = 1, group B: *n* = 1) had developed contralateral CCLR since the last follow-up examination.

### 3.2. Acceptance of Nutraceutical Supplementation

The administration of AS was well tolerated throughout the entirety of the study duration. However, one dog experienced bloody diarrhea beginning on day 3 following TPLO. The AS supplementation was continued and carprofen was temporarily discontinued, leading to the resolution of clinical symptoms within 48 h. Additionally, during the initial phase, three dogs displayed initial hesitance or limited acceptance of AS supplementation, yet this issue was resolved within 2 to 4 days upon the commencement of supplementation.

### 3.3. Gait Analysis

Preoperatively, PVF was not different between the study groups (group A: 29.0 ± 9.2, group B: 30.2 ± 7.9; *p* = 0.6264). On follow-up, PVF was significantly higher for group A compared to group B at every time point (see Table 2). PVF was 40.2 ± 5.0 in group A and 36.8 ± 4.4 in group B at 6 weeks (*p* = 0.0143), while at 12 weeks PVF in group A was 42.5 ± 5.1 and 39.5 ± 4.1 in group B (*p* = 0.0275). At 24 weeks, both groups reached the highest values, with group A still being significantly higher in PVF (group A: 44.2 ± 4.1, group B: 41.4 ± 3.9; *p* = 0.0346).

Preoperatively, dPVF corresponded to a noticeable lameness in both groups (group A: 22.4 ± 13.6, group B: 18.3 ± 10.0; *p* = 0.2297). Six weeks after TPLO, a distinct improvement was achieved in both groups, with significantly smaller inter-limb differences in group A (group A: 4.5 ± 4.1, group B: 8.3 ± 5.6; *p* = 0.01). Also at 12 weeks, group A had significantly lower values than group B (group A: 2.0 ± 2.8, group B: 4.5 ± 3.6; *p* = 0.0114). At that time, group A reached a mean dPVF value in the range of physiological inter-limb difference (<3.2%). After 24 weeks, both groups appeared to have physiologic inter-limb differences (group A: 0.8 ± 1.4, group B: 3.1 ± 2.8; *p* = 0.0008), with group A still showing significantly lower values than group B (see Table 3).

**Figure 2:**
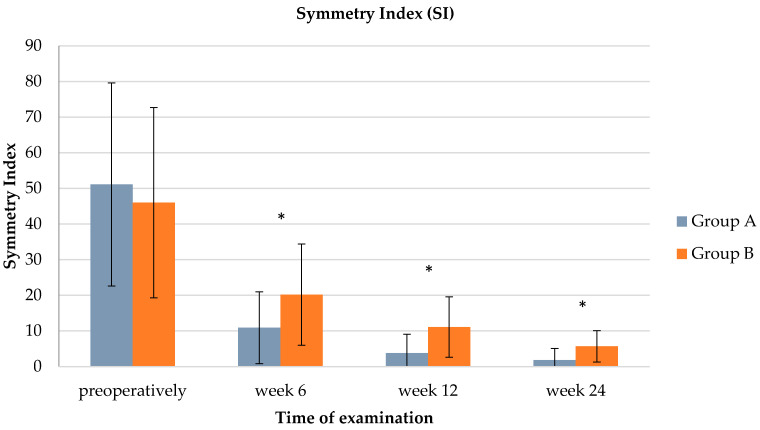
Bar chart illustrating a comparison of the hind limb symmetry index (SI) between groups A and B (preoperatively: *p* = 0.5194, week 6: *p* = 0.0119, week 12: *p* = 0.001, week 24: *p* = 0.0026). Whiskers illustrate the range of the results and * indicates significant group difference.

SI showed a similar pattern, with group A having significantly lower mean SI values compared to group B at every recheck (see Table 4 and Figure 2). Group A regained balanced force distribution (≤9) already at 12 weeks, while group B showed a balanced gait only 24 weeks after TPLO.

Binary coding of dPVF (see Table 5) and SI (see Table 6) allowed patients to be categorized as lame or free of detectable lameness. At the time of initial presentation, all study patients were classified as lame. After 6 weeks, about twice as many dogs in group A gained physiological dPVF compared to group B (group A: 41.7%, group B: 20.8%; *p* = 0.1222). This trend was maintained in the course of further control examinations, so that at week 12, group A still included significantly more lame-free dogs than group B (group A: 80%, group B: 48%; *p* = 0.0241). At 24 weeks, almost all of the dogs in group A had recovered (95.8%), while only 52.9% of the dogs in group B were considered free of detectable lameness, based on dPVF (*p* = 0.0012). Based on binary SI coding, only at week 12 were significantly more dogs in group A considered to be in the physiological range (≤ 9) compared to group B (group A: 87.0%, group B: 41.7%; *p* = 0.0014) (see Table 6). 

### 3.4. LOAD Score

**Figure 3:**
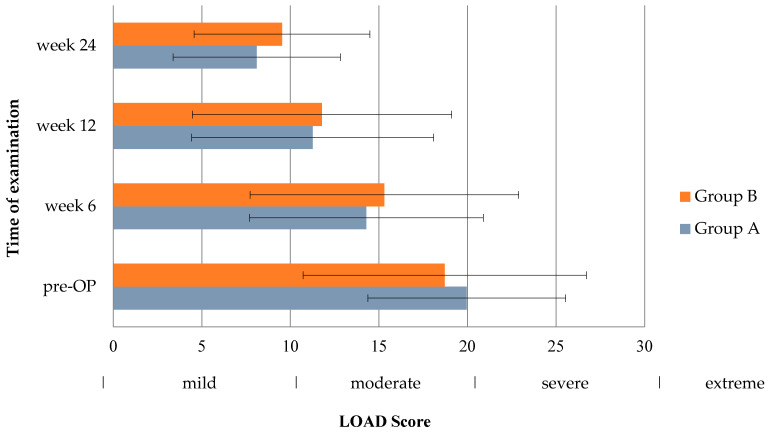
Bar chart showing Liverpool Osteoarthritis in Dogs (LOAD) scores in the inter-group comparison (pre-OP: *p* = 0.859, week 6: *p* = 0.713, week 12: *p* = 1.0, week 24: *p* = 0.576). Whiskers illustrate the range of the results.

Preoperatively, 49 owners completed the LOAD questionnaire (group A = 24, group B = 25). Forty-seven questionnaires were filled out at 6 weeks (group A = 24, group B = 23), forty-six at 12 weeks (group A = 23, group B = 23), and thirty-seven at 24 weeks (group A = 20, group B: n = 17). At inclusion, aggregate LOAD score indicated a moderate lameness in both groups (group A: 20.0 ± 5.6, group B: 18.7 ± 8.0; *p* = 0.859), being close to the next higher category “severe”. In the postoperative phase, LOAD scores declined, but remained in the category “mild” at 6 weeks (group A: 14.3 ± 6.6, group B: 15.3 ± 7.6; *p* = 0.713) and at 12 weeks (group A: 11.3 ± 6.8, group B: 11.8 ± 7.3; *p* = 1.0) for both groups. At 24 weeks following TPLO, dogs had to be considered normal or only mildly affected, based on aggregate LOAD scores of 8.1 ± 4.7 in group A and 9.5 ± 5.0 in group B (*p* = 0.576). At no time point was the aggregate LOAD score significantly different between both groups (see Figure 3).

## 4. Discussion

Our findings clearly indicate a noteworthy enhancement in limb function through the introduction of oral AS supplementation. In particular, significantly higher PVF values were consistently noted, alongside consistently lower dPVF and SI values, across the entire 24-week postoperative period in the cohort that received oral AS supplementation (group A). At the time of every control examination, Group A showed a more balanced load distribution than group B and even a return to normalcy by 12 weeks. After 24 weeks, both groups were considered to be without detectable lameness.

Furthermore, we adopted a binary approach to evaluate the dPVF and SI data, taking into consideration the established cutoff value for a physiological gait pattern. This approach enabled us to not only determine the potential superiority of one study group over the other but also to assess the feasibility of achieving a return to normal limb function and the timeline for such normalization. Based on binary dPVF analysis, approximately twice as many dogs in group A were categorized as free from detectable lameness compared to group B, starting as early as the first recheck, six weeks following TPLO. This substantial difference in the proportion of dogs without lameness in group A versus group B persisted throughout the study, culminating in the final gait analysis at 24 weeks. At this point, nearly all dogs in group A exhibited a normal gait (95.8%), whereas only 52.9% in group B displayed the same. In terms of binary SI analysis, the distinction between the two groups was less pronounced, with a significant increase in the proportion of dogs without lameness in group A compared to group B emerging around week twelve, roughly doubling the ratio. By the 24-week mark, both groups achieved similar proportions of dogs without lameness (group A: 95.8% vs. group B: 82.4%). Overall, the likelihood of returning to normal limb function was significantly higher, approximately twice as high, in the supplement group as early as six weeks post-TPLO when considering dPVF and by week 12 when evaluating SI.

In contrast to the findings from the objective gait analysis, the subjective assessment conducted by the dog owners using the LOAD score did not unveil any apparent advantages resulting from AS supplementation at any assessment time point. This incongruity between the two evaluations might stem from the differing perspectives through which owners perceive their dog’s musculoskeletal function as opposed to the method employed in force plate analysis to quantify limb function and assess gait asymmetry [28]. The variability in owner interpretation and the potential impact of a limited group size on statistical precision could also play a role [37]. It is conceivable that a positive placebo effect might have been expected in the group receiving oral supplementation. However, an inverse scenario is also feasible, wherein owners providing their dogs with the supplementation could hold elevated expectations, potentially leading to disappointment due to the nuanced effect of AS. Consequently, these owners might assign more severe scores to their dogs compared to those who did not anticipate any specific changes in the follow-up period. To validate our findings, further studies involving larger group sizes, blinded owners, and the incorporation of additional questionnaires, possibly focusing on diverse facets of postoperative pain, will be necessary.

Based on the presented findings, AS seems to have positive, objectively measurable, and repeatable effects on rehabilitation of dogs after TPLO surgery. The question of whether the high dosage of T2NDC or a possible synergistic effect by its composition is responsible for these findings cannot be answered by the present study; however, we believe that T2NDC is the main factor responsible for the observed improvement in group A. The exploration of methylsulfonylmethane has been the focus of various studies in humans and especially horses, indicating its pain-reducing potential [38,39,40,41]. However, objective evidence for its use in the treatment of OA and adequate statistical significance are still lacking, rendering its efficacy still a subject of controversy [39,40]. Oral supplementation of hyaluronic acid has also been claimed to yield beneficial effects in diminishing OA pain [42,43]. Furthermore, Bowman et al. suggest that combining hyaluronic acid with other nutraceuticals could hold significant promise, given its potential for synergistic effects [44]. Nevertheless, the absence of objective data analysis leaves a void in establishing evidence for the noteworthy impact of oral hyaluronic acid on joint function or pain reduction. In consequence, the most intriguing constituent of AS is T2NDC in its purest and biologically active configuration: a triple helix. Its uniqueness lies in its high collagen concentration, resulting in an administered dose that is up to 18 times higher than those investigated in prior studies [15,16,17,45]. The influence of T2NDC on joint function and pain levels has been examined and validated in several studies in dogs [15,16,17,20,46]. Currently, it is hypothesized that oral tolerization is induced through a mechanism known as bystander suppression, which necessitates only a small amount of antigen. T2NDC functions as an antigen, presenting itself to the gut-associated lymphoid tissue, thereby eliciting an immune response. Another conceivable mechanism is termed clonal anergy, wherein peripheral lymphocyte tolerance is prompted by the ingestion of a substantial dose of antigen. Through this process, hyperactive Th1 cells transition into a state of non-reactivity, modulating the immune system and altering it to prevent self-destruction [15]. Nonetheless, the precise effective daily dosage of orally administered T2NDC required to achieve this state remains undisclosed.

The lack of a pure T2NDC preparation in this study is a limitation that must be considered. Oral administration of only T2NDC in its purest form could have crystallized the effect of collagen. Thus, it remains questionable whether the acceptance, absorption mechanisms, and immunomodulatory properties would have emerged to the same extent with the administration of pure T2NDC. The manufacturers of AS aimed at creating a composition that would lead to synergistic effects of the individual components and to an increased readiness for penetration and thus immune response. Another limitation is the lack of a placebo, which might have enhanced the results obtained [47]. An attempt was made to counteract this limitation by choosing a prospective and randomized study design.

## 5. Conclusions

In summary, our study provides compelling evidence that oral AS supplementation positively influences limb function following TPLO surgery in dogs. This effect becomes particularly evident in the short to mid-term postoperative phase. Essentially, the inclusion of AS supplementation is likely to result in a notable reduction of the postoperative recovery period by at least 12 weeks following TPLO surgery.

## Figures and Tables

**Table 1:** Comparative descriptive statistics of study groups.

	Group A (*n* = 25)	Group B *(n* = 25)	*p*-Value
Age (years)	5.52 ± 2.62	6.12 ± 2.83	0.44
Weight (kg)	35.24 ± 6.16	32.9 ± 6.5	0.21
Sex			1.0
Male	9	9	
Female	16	16	
Body condition score (1–9)	5 ± 0.58	5 ± 0.54	0.8
Subtotal meniscectomy	8	10	0.57
Cruciate tear type			0.69
Partial	4	3	
Complete	21	22	
CCL side			1.0
Left	15	15	
Right	10	10	

Note: Values are presented as means ± SD. *p* ≤ 0.05 is considered significant.

**Table 2:** Results of peak vertical force (PVF), giving the mean values ± standard deviation.

	Time			
	Pre-OP (n = 49)	Week 6 (n = 48)	Week 12 (n = 47)	Week 24 (n = 41)
Group A	29.0 ± 9.2	40.2 ± 5.0	42.5 ± 5.1	44.2 ± 4.1
Group B	30.2 ± 7.9	36.8 ± 4.4	39.5 ± 4.1	41.4 ± 3.9
*p*-value	0.6264	0.0143	0.0275	0.0346

Note: *p* ≤ 0.05 is considered significant.

**Table 3:** Results of difference in peak vertical force (dPVF) between the operated and unaffected hind limb, giving the mean values ± standard deviation.

	Time			
	Pre-OP (*n* = 49)	Week 6 (*n* = 48)	Week 12 (*n* = 47)	Week 24 (*n* = 41)
Group A	22.4 ± 13.6	4.5 ± 4.1	2.0 ± 2.8	0.8 ± 1.4
Group B	18.3 ± 10	8.3 ± 5.6	4.5 ± 3.6	3.1 ± 2.8
*p*-value	0.2297	0.01	0.0114	0.0008

Note: *p* ≤ 0.05 is considered significant.

**Table 4:** Results of the hind limb symmetry index (SI), giving the mean values ± standard deviation.

	Time			
	Pre-OP (*n* = 49)	Week 6 (*n* = 48)	Week 12 (*n* = 47)	Week 24 (*n* = 41)
Group A	51.1 ± 28.5	10.9 ± 10.1	3.8 ± 5.3	1.8 ± 3.3
Group B	46.0 ± 26.7	20.2 ± 14.2	11.1 ± 8.5	5.7 ± 4.4
*p*-value	0.5194	0.0119	0.001	0.0026

Note: *p* ≤ 0.05 is considered significant.

**Table 5:** Ratio of physiological difference in peak vertical force (dPVF).

	Time			
	Pre-OP (*n* = 49)	Week 6 (*n* = 48)	Week 12 (*n* = 47)	Week 24 (*n* = 41)
Group A	0	41.7	80.0	95.8
Group B	0	20.8	48.0	52.9
*p*-value	-	0.1222	0.0241	0.0012

Note: *p* ≤ 0.05 is considered significant.

**Table 6:** Ratio of physiological hind limb symmetry index (SI).

	Time			
	Pre-OP (*n* = 49)	Week 6 (*n* = 48)	Week 12 (*n* = 47)	Week 24 (*n* = 41)
Group A	0	41.7	87.0	95.8
Group B	0	20.8	41.7	82.4
*p*-value	-	0.1222	0.0014	0.1594

Note: *p* ≤ 0.05 is considered significant.

## Data Availability

The data presented in this study are available on request from the corresponding author. The data are not publicly available due to national data security restrictions.
